# Tracheal Dysplasia Precedes Bronchial Dysplasia in Mouse Model of N-Nitroso Trischloroethylurea Induced Squamous Cell Lung Cancer

**DOI:** 10.1371/journal.pone.0122823

**Published:** 2015-04-10

**Authors:** Moumita Ghosh, Lori D. Dwyer-Nield, Jennifer B. Kwon, Lea Barthel, William J. Janssen, Daniel T. Merrick, Robert L. Keith

**Affiliations:** 1 Department of Pediatrics, National Jewish Health, Denver, Colorado, United States of America; 2 Department of Pharmaceutical Sciences, University of Colorado, Aurora, Colorado, United States of America; 3 Department of Medicine, National Jewish Health, Denver, Colorado, United States of America; 4 Department of Medicine, Division of Pulmonary Sciences and Critical Care Medicine, Denver Veteran Affairs Medical Center, Denver, Colorado, United States of America; 5 Department of Pathology, University of Colorado, Aurora, Colorado, United States of America; Cincinnati Children's Hospital Medical Center, UNITED STATES

## Abstract

Squamous cell lung cancer (SCC) is the second leading cause of lung cancer death in the US and has a 5-year survival rate of only 16%. Histological changes in the bronchial epithelium termed dysplasia are precursors to invasive SCC. However, the cellular mechanisms that cause dysplasia are unknown. To fill this knowledge gap, we used topical application of N-nitroso-tris chloroethylurea (NTCU) for 32 weeks to induce squamous dysplasia and SCC in mice. At 32 weeks the predominant cell type in the dysplastic airways was Keratin (K) 5 and K14 expressing basal cells. Notably, basal cells are extremely rare in the normal mouse bronchial epithelium but are abundant in the trachea. We therefore evaluated time-dependent changes in tracheal and bronchial histopathology after NTCU exposure (4, 8, 12, 16, 25 and 32 weeks). We show that tracheal dysplasia occurs significantly earlier than that of the bronchial epithelium (12 weeks vs. 25 weeks). This was associated with increased numbers of K5^+^/K14^+^ tracheal basal cells and a complete loss of secretory (Club cell secretory protein expressing CCSP^+^) and ciliated cells. TUNEL staining of NTCU treated tissues confirmed that the loss of CCSP^+^ and ciliated cells was not due to apoptosis. However, mitotic index (measured by bromodeoxyuridine incorporation) showed that NTCU treatment increased proliferation of K5^+^ basal cells in the trachea, and altered bronchial mitotic population from CCSP^+^ to K5^+^ basal cells. Thus, we demonstrate that NTCU-induced lung epithelial dysplasia starts in the tracheal epithelium, and is followed by basal cell metaplasia of the bronchial epithelium. This analysis extends our knowledge of the NTCU-SCC model by defining the early changes in epithelial cell phenotypes in distinct airway locations, and this may assist in identifying new targets for future chemoprevention studies.

## Introduction

Squamous cell lung cancer (SCC) is the second most common type of lung cancer and accounted for approximately 40,000 deaths in the United States in 2013 [1]. The 5-year survival rate for SCC is only 16%, a profoundly disappointing statistic [1]. Preneoplastic bronchial dysplasias are the first detectable histological markers of SCC [2, 3], and histologic improvement in these lesions serve as end points in SCC chemoprevention trials [4, 5]. However, the processes leading to dysplasia are poorly understood. The goal of this study is to determine the sequence of cellular changes that leads to squamous dysplasia, the precursor to SCC.

This effort requires a mouse model of SCC that faithfully recapitulates the human disease. There are three established murine models of lung SCC: 1) topical treatment with N-nitroso tris chloroethylurea (NTCU) [6–9]; 2) inactivation of tumor suppressor LKB1 [10]; and 3) downregulation of IKKα [11]. Importantly, NTCU exposure is the only model that generates squamous dysplasia of the mouse bronchial epithelium that is pathologically equivalent to the dysplasia encountered in human smokers [6]. Dose and time dependent generation of high-grade dysplasia and SCC makes the NTCU model an optimal system to investigate early phenotypic changes in central airway epithelial cells during dysplasia development.

The mammalian respiratory epithelium is divided into the tracheal, bronchial, bronchiolar and alveolar regions [12]. In humans, the basal cell containing pseudostratified epithelium extends from the trachea through the terminal bronchiole. In contrast, this pseudostratified epithelium is largely restricted to the trachea in mice (S1 Fig), and the epithelium transitions to a simple columnar epithelium *lacking basal cells* in the mainstem bronchi [13]. The normal bronchial epithelium consists of secretory cells that are defined by the expression of Club cell secretory protein (CCSP^+^) and ciliated cells defined by motile cilia that express acetylated tubulin (ACT^+^). This epithelium lacks Keratin (K) 5/14 expressing basal cells [13, 14]. Therefore the appearance of basal cells in the mouse bronchial epithelium is abnormal and is termed basal cell metaplasia [13].

In order to investigate the mechanisms leading to epithelial dysplasia we evaluated the effects of NTCU treatment on the tracheal and bronchial regions of the mouse airways by performing a time-course analysis. Tracheal dysplasia was detected between 8–12 weeks of NTCU exposure. This was characterized by increased numbers of K5^+^, K14^+^ and p63 expressing basal cells, loss of CCSP^+^ and ACT^+^ cells, increased basal cell proliferation, and expression of the squamous differentiation marker involucrin. Bronchial dysplasia was first observed at 25 weeks and was associated with basal cell metaplasia and replacement of the Club cell mitotic pool by highly proliferative basal cells. Based on these findings we conclude that NTCU-induced phenotypic changes in the tracheal epithelial cells occur prior to bronchial dysplasia and SCC.

## Materials and Methods

### Materials

Detailed information about materials used in the study is described in the supporting information section (S).

#### Animals

Female FVB/n mice were purchased from the Harlan Laboratories at 8 weeks of age.

#### Ethics Statement

Animals were maintained at the Denver Veteran Affairs Medical Center in a pathogen free facility. This study was carried out in strict accordance with the recommendations in the Guide for the Care and Use of Laboratory Animals of the National Institutes of Health. The protocol was reviewed and approved by Denver Veteran Affairs Medical Center Animal Care and Use Committee (Approval number: MR1304M). All surgery was performed under sodium pentobarbital anesthesia, and all efforts were made to minimize suffering.

### Methods

#### Administration of NTCU

The FVB/n strain was selected for its known susceptibility to NTCU-induced SCC [9]. Topical NTCU application was initiated at 10 weeks of age and occurred twice a week for a maximum of 32 weeks at a concentration of 20 mM/L NTCU in acetone. Twenty-five μl NTCU was applied to the shaved dorsal skin. Control mice received acetone vehicle alone. At 4, 8, 12, 16, 25, and 32 weeks lung and trachea/esophagus were insufflated, preserved in formalin, and embedded in paraffin for histological and immunofluorescent analysis.

To determine the mitotic index of epithelial cells, the nucleotide analogue bromodeoxy uridine (BrdU) was dissolved at 5 mg/ml in saline and was delivered at 10 μl/g body weight intraperitoneally 2 hours prior to tissue harvest.

#### Histology and immunofluorescent analysis

Tissue sections (5 μm) from the trachea and the right lower lobe of lung were generated from paraffin embedded tissue. Sections were stained with hematoxylin and eosin. The study pathologist (DTM) graded the levels of dysplasia and performed all the histological analyses.

For immunofluorescence studies, antigens were detected with antibodies to K5, K14, CCSP, ACT, BrdU, p63, NGFR, and involucrin employing previously described staining methods [15]. Images were acquired using an upright Zeiss Imager Z1 fluorescent microscope and AxioVision software (Carl Zeiss).

#### TUNEL staining to identify apoptotic cells

TUNEL staining of NTCU treated tracheal and bronchial epithelium was performed using DeadEnd^TM^ Fluorometric TUNEL system from Promega following manufacturer’s instructions. Positive controls for TUNEL staining were generated by treating the tissue section with DNAse.

#### Morphometric analysis

Changes in frequency of tracheal epithelial cell types were analyzed using morphometric methods [15, 16]. A total of 9 areas per trachea were randomly selected and imaged (200x magnification). Briefly, volume densities (Vv) were calculated by counting the number of points intersecting the structure of interest divided by the total points intersecting the reference space using a grid with evenly spaced points. Surface density (Sv) was calculated by counting the number of intersections between a cycloid grid and the basement membrane divided by the length of the test line within the epithelium. The Vv/Sv values represent the volume of a specific cell type per unit area of the basement membrane [17].

#### Statistical Analysis

Number of mice used for each experiment is provided in the results and/or in the figure legends. Data were analyzed using GraphPad Prism v. 6.01 software. The statistical analysis used *t* tests and one-way ANOVA with post analysis by the Bonferroni multiple comparison method.

## Results

### 32-week NTCU treatment induces high-grade bronchial dysplasia and lung SCC

**Fig 1:**
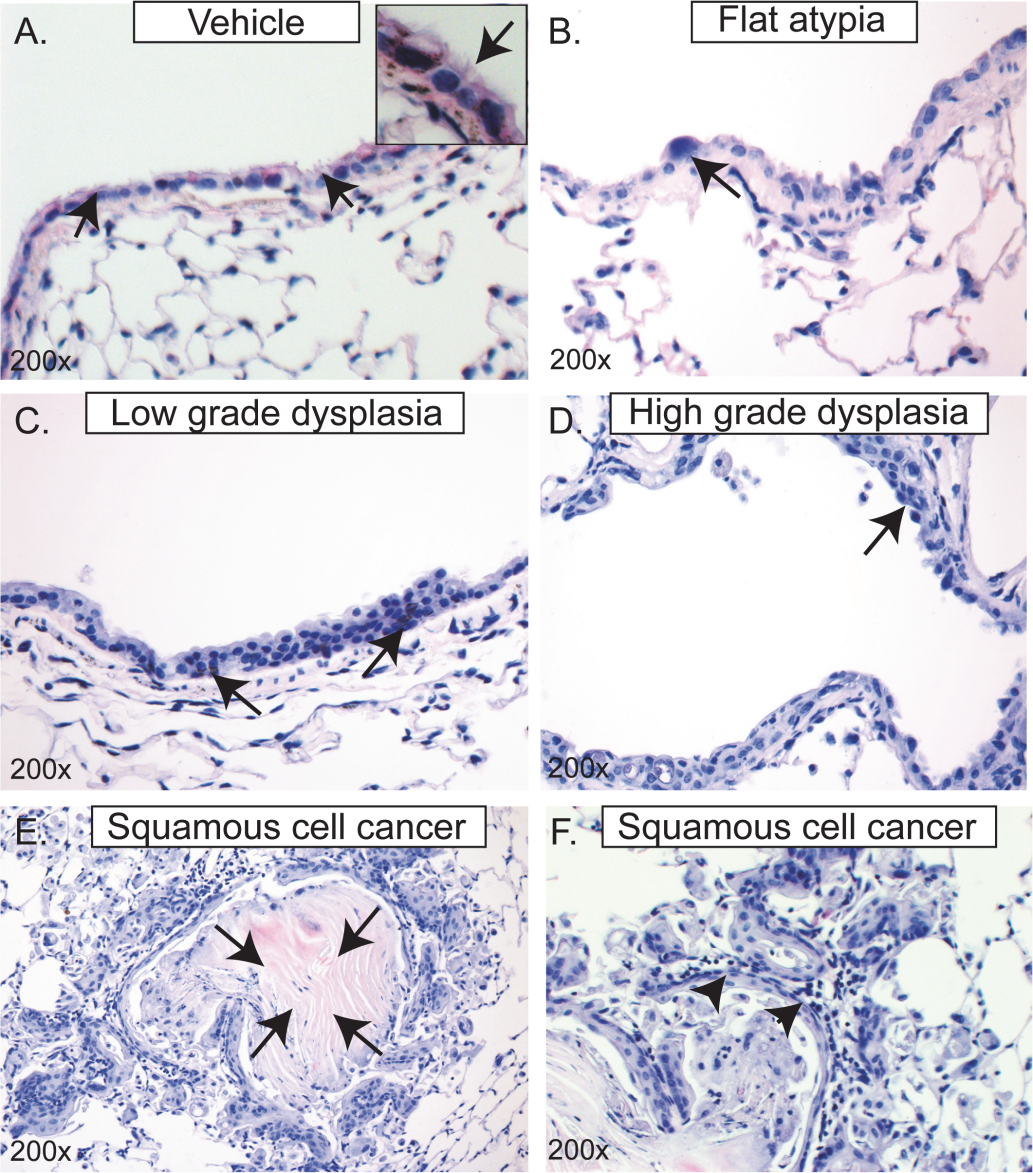
32-week NTCU treatment induces high-grade bronchial dysplasia and lung SCC. H&E stained images of **(A)** Normal epithelium (acetone vehicle), and NTCU-induced **(B-D)** premalignant lesions and **(E&F)** invasive carcinoma. **(A)** Normal epithelium: basally located nuclei and apical cilia (arrows and inset). **(B)** Flat-atypia: single cell layer, but enlarged hyperchromatic nuclei (arrow) and infrequent loss of cilia. **(C)** Low-grade dysplasia: stratified non-ciliated epithelium, horizontal orientation of nuclei and decreased nuclear:cytosolic ratio. **(D)** High-grade dysplasia: stratified squamous non-ciliated epithelium with lack of orientation and increased nuclear:cytosolic ratio. **(E)** Lung SCC: invasive squamous cell carcinoma with arrows showing central keratinaization in the nest of the tumor. **(F)** SCC is generated from the high-grade dysplastic airways (arrow-heads). Original magnifications are indicated in each panel. The pictures shown here are the representative images from 10 mice each for vehicle and NTCU group.

We previously reported that bi-weekly topical application of 40 mM NTCU in FVB/n mice for 32 weeks caused a series of endobronchial squamous lesions classified as flat atypia, low and high grade dysplasia, as well as invasive SCC [6]. Because 40 mM NTCU treatment caused high mortality, mice were treated with 4, and 8 mM NTCU and histopathology of bronchial epithelium was examined. These lower doses were well tolerated but pathology was largely limited to flat atypia [6]. In contrast, application of 20 mM NTCU twice a week for 32 weeks (used in this study) resulted in all grades of bronchial dysplasia and lung SCC without significant mortality (Fig 1 & S2 A and S2 B Fig).

We confirmed that the tumors generated by 20 mM NTCU were SCC by immunofluorescent detection of lung SCC markers (positive for K5, K14, p63, and negative for thyroid transcription factor 1) (S2 C–S2 F Fig). High-grade dysplasias were detected more frequently in the animals that developed invasive SCC. These lesions were observed in the bronchial epithelium and were limited to the larger airways (S2 G and S2 H Fig).

### Molecular phenotype of the cells present in NTCU-induced bronchial dysplasia

**Fig 2:**
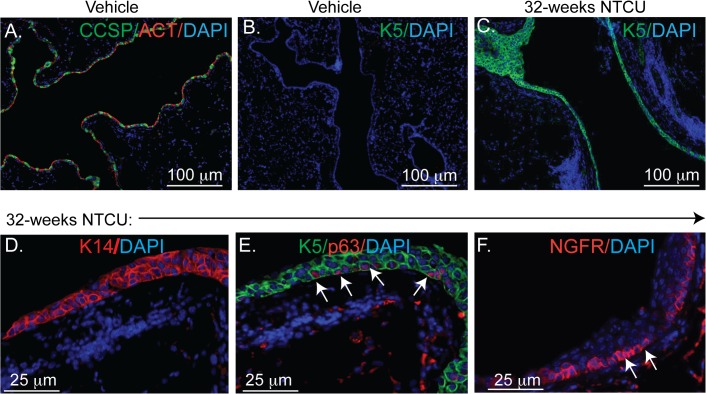
Molecular phenotype of the cells present in NTCU-induced bronchial dysplasia. Bronchial epithelium from mice treated for 32 weeks with vehicle **(A-B)** or NTCU **(C-F).** Tissues were stained with antibodies against **(A)** CCSP (green), ACT (red) and **(B & C)** K5 (green). Expression of other basal cell markers was detected by staining with **(D**) K14 (red), **(E)** p63 (red) and K5 (green), and **(F)** nerve growth factor receptor (NGFR, red). DAPI staining (blue) in all images depicts nuclei. Representative images from 10 mice treated with vehicle or NTCU.

To investigate the molecular phenotype of the cells that populated dysplastic bronchial epithelium, we compared the frequency of basal, Club, and ciliated cells in mice that were treated for 32 weeks with vehicle or NTCU (n = 10 mice/group). The bronchial epithelium of vehicle treated mice contained CCSP^+^ and ACT^+^ cells and lacked K5^+^ basal cells (Fig 2 A and 2 B).

In contrast, the epithelium of NTCU treated mice contained K5^+^ basal cells (Fig 2C). To investigate whether cells within dysplastic regions expressed other basal cell markers, 32 week sections were stained with K14, the basal cell-specific transcription factor p63, and nerve growth factor receptor (NGFR) [18] (Fig 2 D–2 F). These analyses showed that chronic NTCU treatment caused the appearance of cells that expressed multiple basal cell markers in the bronchial epithelium.

To determine whether appearance of basal cells in the dysplastic bronchial epithelium coincides with the loss of normal bronchial epithelial cells, we immunostained adjacent serial sections of lung tissues for K5, CCSP or ACT (n = 10 sections/mouse). These analyses revealed that epithelial dysplasia is associated with the appearance of K5^+^ basal cells and loss of CCSP^+^ and ACT^+^ cells (S3 A and S3 B Fig). These studies indicate that the bronchial dysplasia involves basal cell metaplasia and depletion of resident Club and ciliated cells.

### Development of bronchial dysplasia over time

**Fig 3:**
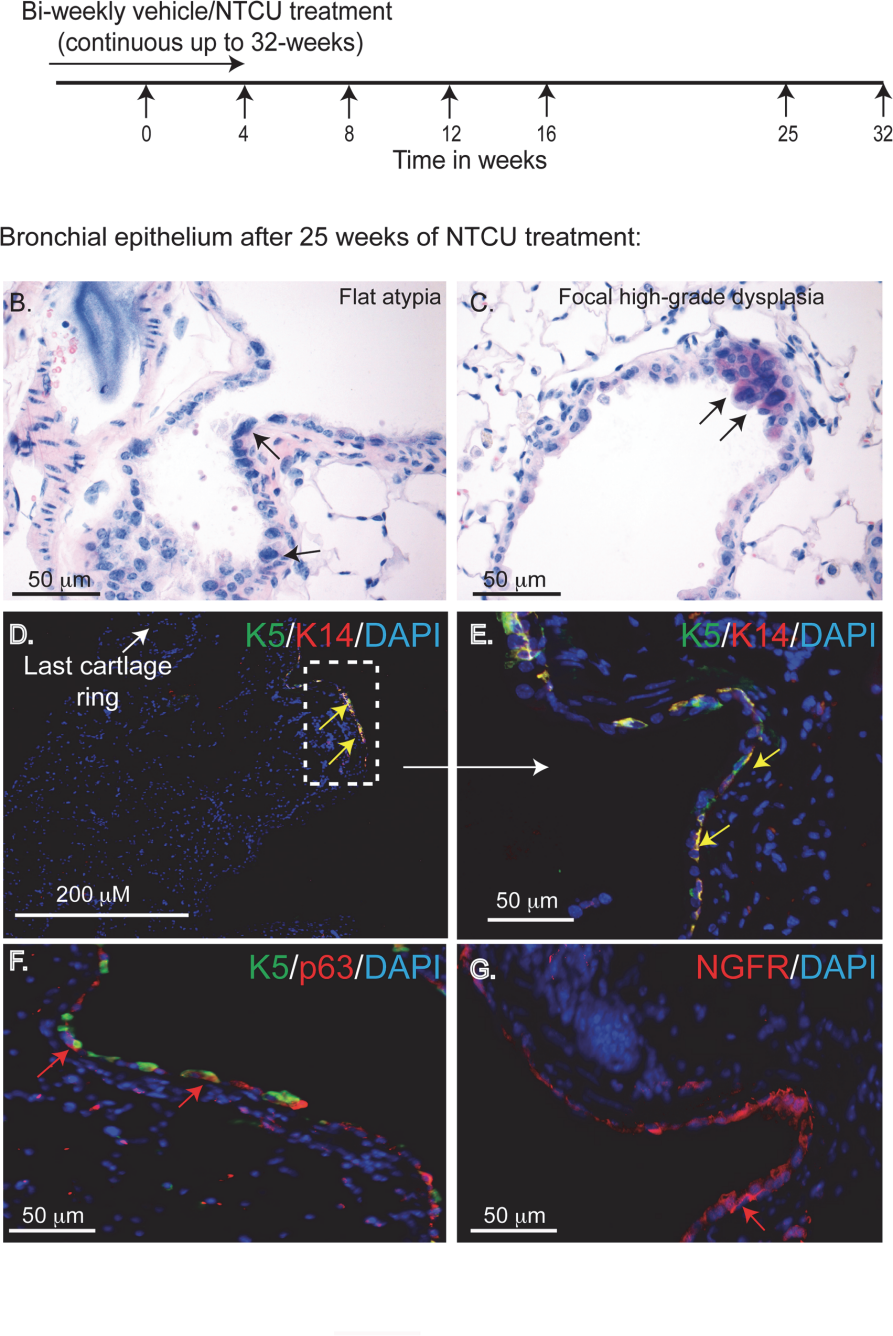
Development of bronchial dysplasia over time. **(A)** Study design for time-course analysis of NTCU exposure. Arrows indicate the time-points when tissues were collected. **(B-C)** H & E stained bronchial tissues after 25 weeks of NTCU treatment. Immunofluorescence staining of dysplastic bronchial epithelium **(D-G)**. **(E)** Amplified image of the boxed area shown in **(D).** Antigens used and scale bars are mentioned in the respective panel. Representative images are from 5 NTCU treated mice.

To investigate the sequence of events that lead to NTCU-induced changes in the bronchial epithelium we conducted a time-course analysis. Mice were exposed to NTCU for 4, 8, 12, 16, 25, or 32 weeks and histopathology of the bronchial epithelium was analyzed (Fig 3A).

Surprisingly, no dysplastic change was observed until 25 weeks of exposure. At this time different grades of squamous lesions, ranging from flat atypia to focal high-grade dysplasia, were detected in the bronchial epithelium (Fig 3 B and 3 C). Significantly, the development of bronchial dysplasia also coincided with the first appearance of cells expressing basal cell markers (K5^+^, K14^+^, p63 and NGFR) in the dysplastic regions (Fig 3 D–3 G, arrow in D shows the distal tracheal epithelium). These changes were progressive and at 32 weeks K5^+^ cells populated the entire bronchial epithelium (S4 A and S4 B Fig).

### NTCU treatment caused squamous dysplasia of the tracheal epithelium

**Fig 4:**
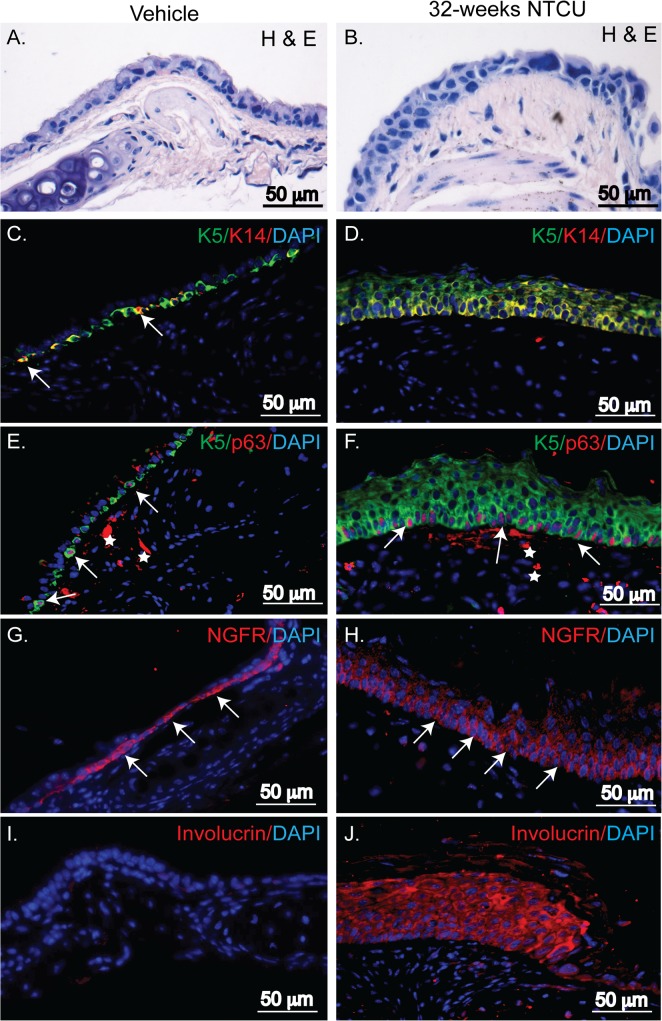
NTCU induced squamous dysplasia of the tracheal epithelium: Tracheal epithelium of vehicle (left column) and NTCU treated (right column) mice at 32 weeks. **(A&B)** H&E stained picture showing high-grade dysplasia of the tracheal epithelium induced by NTCU. **(C&D)** Dual staining with K5 (green) and K14 (red), **(E&F)** K5 (green) and p63 (red) and arrows show dual positive cells. Nonspecific staining under the epithelium are shown by white * symbol. **(G&H)** Staining for nerve growth factor receptor NGFR and **(I &J)** squamous differentiation marker, involucrin. DAPI staining (blue) in all images indicates nuclei and scale bar is indicated in each panel. Representative images from 10 mice each for vehicle and NTCU group.

Since basal cells are not normally found in the murine bronchial epithelium (Fig 2B), we next evaluated the impact of NTCU treatment in the basal cell containing tracheal epithelium. Low-grade tracheal dysplasia was observed at 4 weeks and the extent of the dysplasia became more extensive with longer exposure times (S5 A–S5 D Fig). Comparison of tracheal histopathology between 32 weeks vehicle and NTCU treated mice (n = 10 mice/group) showed high-grade dysplasia of the tracheal epithelium (Fig 4 A and 4 B). Similar to bronchial dysplasia, this was characterized by a non-ciliated epithelium and the presence of cells with horizontally oriented nuclei and increased nuclear-cytosolic ratio. At the molecular level, NTCU treatment increased the frequency of K5, K14, p63, and NGFR expressing basal cells in the trachea (Fig 4 C, 4 E and 4 G vs. 4D, 4F and 4 H). To determine whether NTCU treatment induced squamous differentiation of the tracheal epithelium, we stained tracheal tissues with involucrin, a marker for stratified squamous epithelia [19, 20]. High-level expression of involucrin confirmed that the pseudostratified tracheal epithelium had transformed into a stratified squamous epithelium (Fig 4 I and 4 J). These results illustrate that tracheal epithelial cells respond to NTCU treatment much earlier than the bronchial epithelial cells.

### Tracheal dysplasia was associated with time-dependent increase in K5, K14, p63 and NGFR-expressing basal cells and decrease in CCSP^+^ and ACT^+^ cells

**Fig 5:**
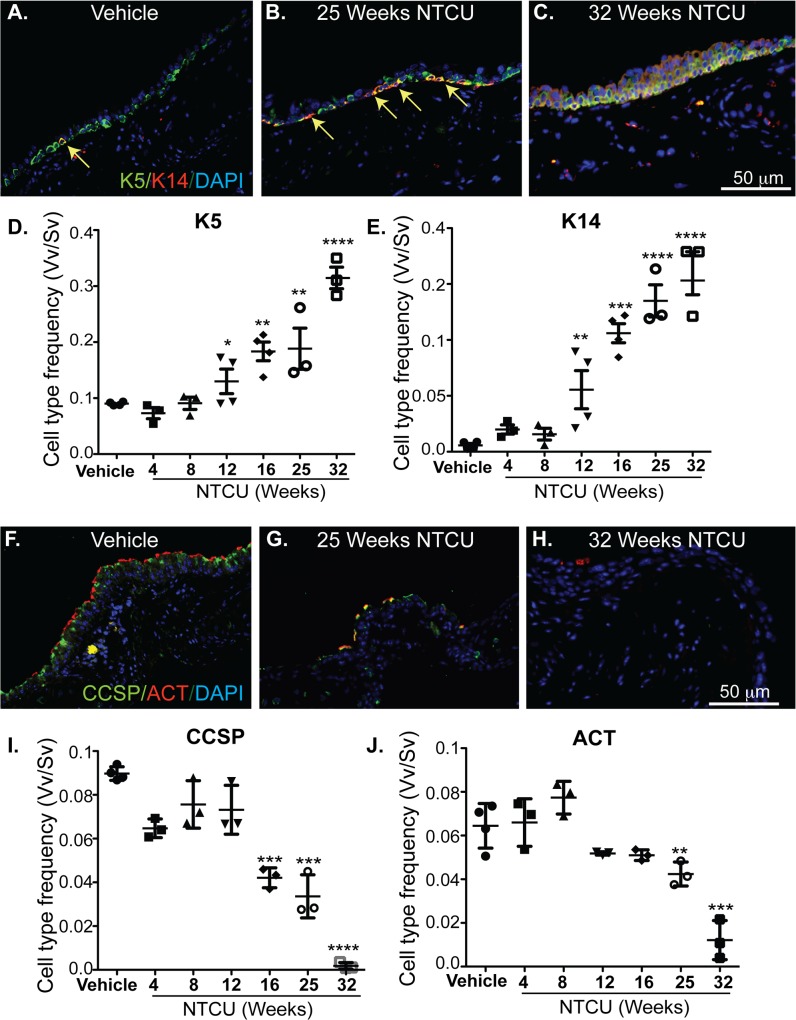
NTCU-induced increase in K5 and K14 expressing basal cells and decrease in CCSP and ACT positive cells in the tracheal epithelium. **(A-C)** Immunostaining of the tracheal epithelium with antibodies to K5 (green), K14 (red) and DAPI (blue). Yellow arrows depict K5/K14-dual positive cells. **(D&E)** Quantification of K5, K14 cell frequency over time using morphometric analyses. **(F-H)** Immunostaining of the tracheal epithelium against antibodies to CCSP (green), ACT (red), DAPI (blue) and **(I&J)** quantification of CCSP and ACT positive cell frequency. Each point in the graphs D, E, I & J represent individual mouse, and n = 3–4 mice were used for each time-point. Vv/Sv represents the volume density of specific cell types (Vv) as a function of surface density (Sv) with the epithelium used as the reference volume. 1-Way ANOVA and post test using Bonferroni multiple comparison methods were used for statistical analysis. Significant difference (p<0.05) between the vehicle control and other comparison groups are shown by stars (****, p<0.0001, ***, p<0.001, ** p<0.01, and * p<0.05).

Basal cells comprise 30% of all epithelial cells present in the trachea and express K5 [15, 21, 22]. In vehicle treated epithelium, a subset of K5^+^ cells (20%) co-express K14 and a smaller subset (7–10%) co-express p63 (Fig 4 C and 4 E). In contrast, tracheal basal cells uniformly express NGFR (Fig 4G). To determine whether NTCU exposure causes a time-dependent increase in basal cell subsets we stained tracheal sections for the basal cell markers described above. Morphometric analysis [15, 16] of tracheal tissues (n = 3–4 trachea/time point) revealed a significant (p < 0.05) increase in number of both K5^+^ and K14^+^ cells starting 12-weeks after initiation of NTCU treatment (Figs 5 A–4 E). Similar increases were observed for both p63^+^ (p < 0.05 at 12 weeks) and NGFR^+^ (p < 0.05 at 16 weeks) basal cells (S6 Fig A and S6 B).

Quantification of tracheal CCSP^+^ and ACT^+^ cells demonstrated time-dependent loss of these cell types after NTCU treatment (Fig 5 F–5 J). We used TUNEL staining of tracheal epithelium to investigate whether the decrease in CCSP^+^ and ACT^+^ cells was associated with increased apoptosis. We did not detect an increase in TUNEL^+^ cells in the tracheal epithelium at any time point after NTCU exposure suggesting the decrease in CCSP^+^ and ACT^+^ cells is not due to increased apoptosis (S7 A–S7 D Fig). Importantly, a significant increase (p<0.05) in total epithelial cell numbers or epithelial hyperplasia was only detected at 32-week time (S6 C Fig). These results suggest that at the early time-points the loss of secretory and ciliated cells was balanced by an increase in the numbers of basal cells.

### Mitotic index of tracheal epithelial cells in NTCU-induced dysplasia

**Fig 6:**
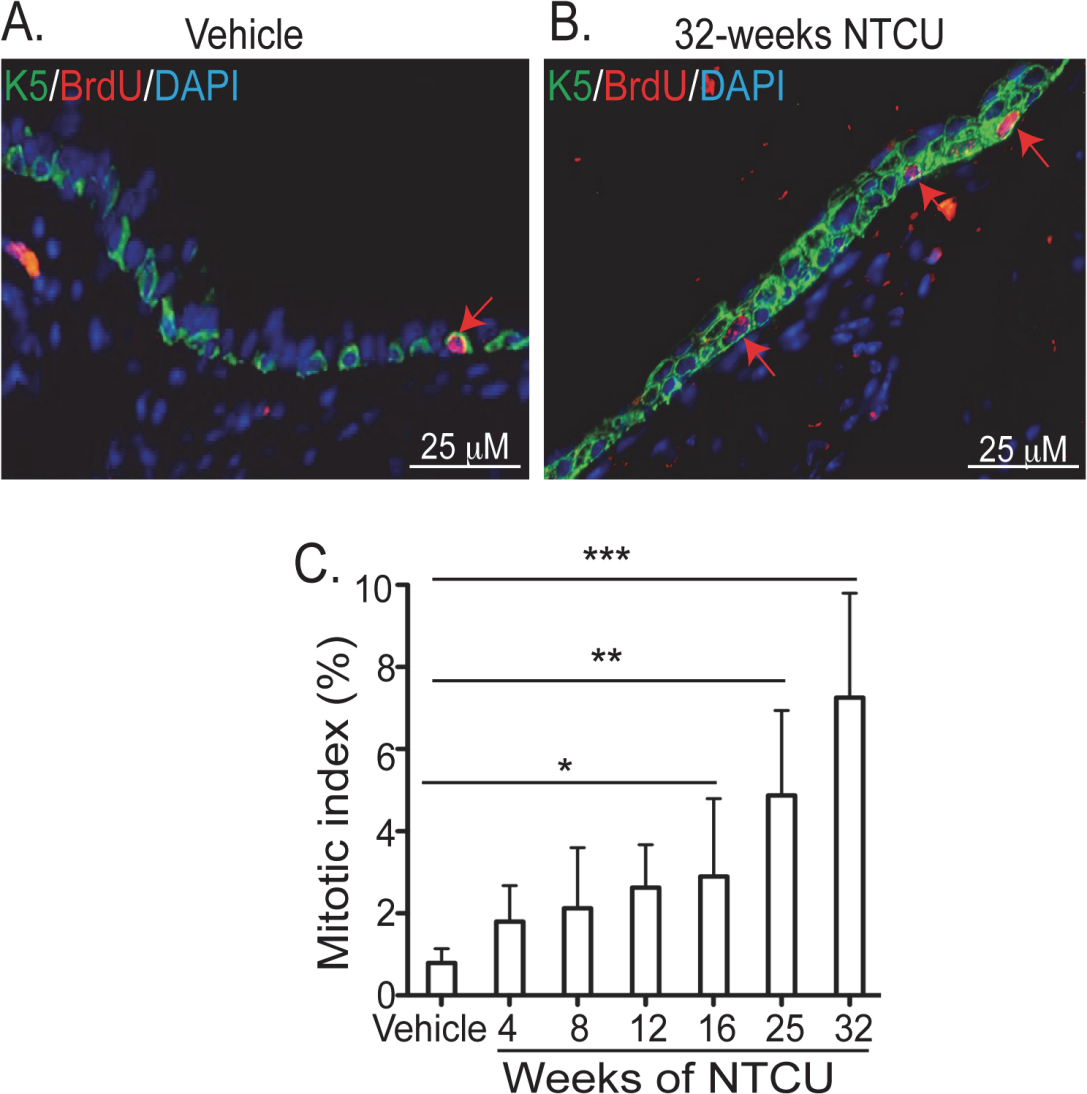
NTCU treatment increased the mitotic index of tracheal basal cells. **(A-B**) Tracheal tissue sections from mice treated with vehicle or NTCU for 32 weeks were stained for K5 (green), BrdU (red) and DAPI (blue). Representative pictures from n = 4 mice per group was used. **(C)** Quantification was performed by counting BrdU^+^ nuclei throughout the trachea from proximal to distal axis and represented as % of DAPI^+^ nuclei. Statistical analyses were performed as described in Fig 5.

Analysis of human dysplasia shows that increasing dysplasia grade positively correlates with increased airway epithelial cell proliferation (as measured by Ki-67 positivity) [23, 24]. We used the nucleotide analogue BrdU to determine if NTCU-induced tracheal dysplasia correlates with increased epithelial cell proliferation, (n = 4 tracheas/time point/group). In vehicle treated mice only 0.79 ± 0.34% cells were BrdU^+^ (Fig 6A), demonstrating low turnover rate of the tracheal epithelium in the absence of injury [25].

Importantly, 82.35 ± 7.63% of BrdU^+^ cells co-express K5, which indicated that basal cells are the major mitotic cell type in the tracheal epithelium. Chronic NTCU exposure led to a time-dependent increase in the numbers of BrdU^+^ cells with a 3.5-fold (p<0.05) increase at 16 weeks, 6-fold increase at 25 weeks (p<0.001), and a 10-fold (p<0.001) increase at 32 weeks (Fig 6 B–6 C). These results demonstrated that repeated exposure to NTCU increases proliferation of tracheal basal cells.

### Mitotic index of bronchial epithelial cells after NTCU treatment

**Fig 7:**
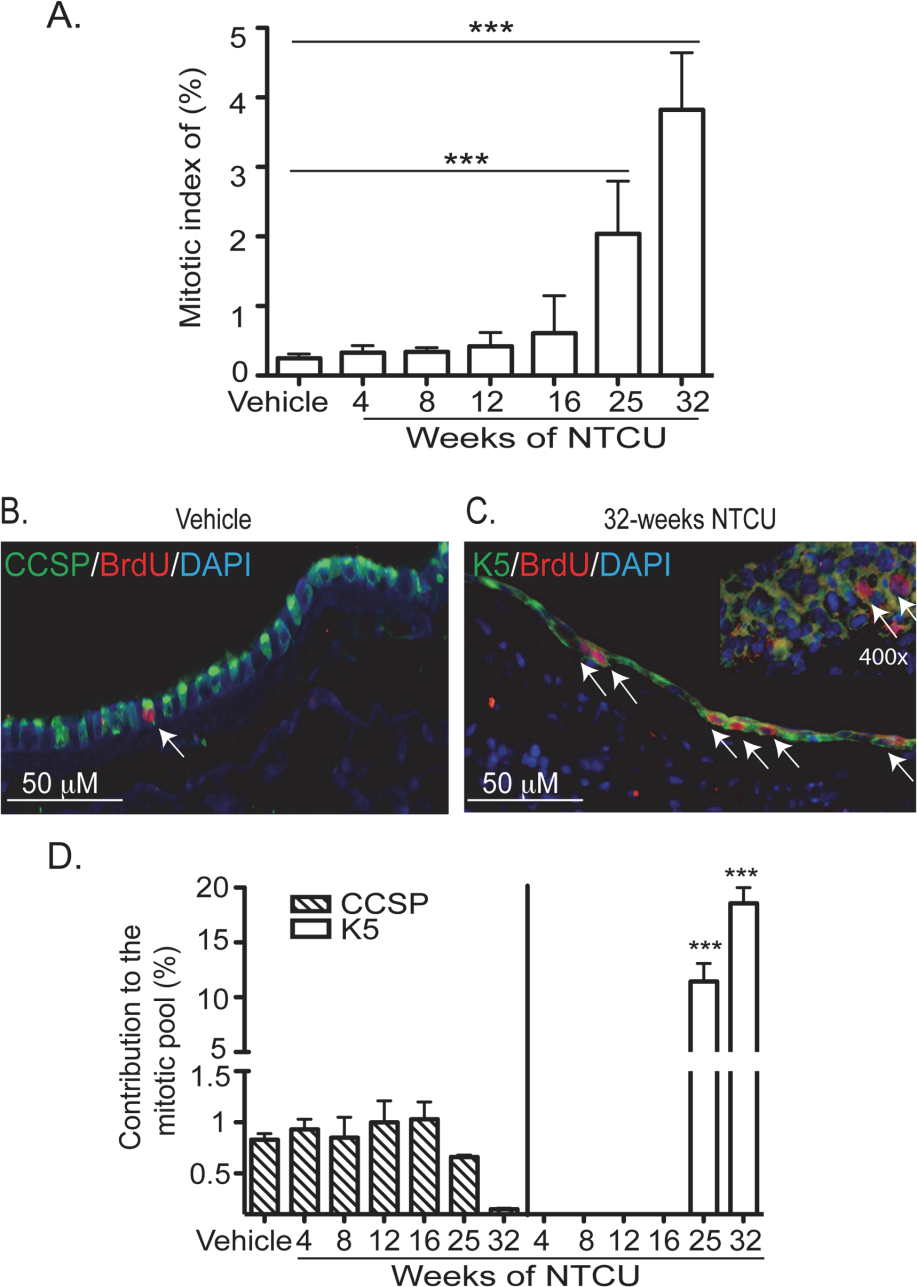
NTCU treatment increased the mitotic index of bronchial epithelial cells. **(A)** Mitotic index of bronchial epithelial cells was measured by counting the numbers of BrdU^+^ nuclei as a % of DAPI^+^ nuclei (all epithelial cells). Quantification was limited to the main axial pathway and the images were taken at 200x magnification from 6 areas of the epithelium per tissue. **(B)** Lung tissues from mice treated with vehicle for 32 weeks were stained for CCSP (green), BrdU (red), and DAPI (blue). **(C)** 32 weeks NTCU treated tissues stained with K5 (green), BrdU (red), and DAPI (blue). Arrows in **B** & **C** show the BrdU^+^ nuclei. Inset in **C** shows the presence of BrdU^+^ cells in a hyperplastic area **(D)** Contribution of each of CCSP and K5 expressing cells in the mitotic pool was quantified as a percent of CCSP-BrdU dual positive/CCSP^+^ cells or K5-BrdU dual positive/K5^+^ cells. Statistical analyses were performed as described in Fig 5.

Quantification of BrdU^+^ cells in the bronchial epithelium of vehicle treated mice (n = 4) showed that only 0.25 ± 0.06% of cells were BrdU^+^. These results support previous reports that steady state turnover rate of bronchial epithelium is slower than that of the tracheal epithelium [25–27]. However, in contrast to the trachea, the mitotic index of bronchial epithelial cells did not change significantly until 25 weeks of NTCU exposure. At 25 weeks frequency of BrdU^+^ cells was 2.04 ± 0.65%, an 8-fold (p<0.01) increase in mitotic index. The mitotic index after 32 weeks NTCU treatment was 3.82 ± 0.72%, an increase over vehicle treated mice of 15-fold (p<0.001) (Fig 7A). However this proliferation rate remained much lower than that observed in the trachea.

To identify the mitotic cell type in the bronchial epithelium after NTCU treatment we co-detected BrdU and the cell type specific markers CCSP or K5. These analyses showed that 100% of mitotic cells in the vehicle treated mice, as well as mice treated with NTCU from 4–16 weeks; co-express CCSP (Fig 7B). These data agree with previous reports that CCSP^+^ cells are the major mitotic cell type in murine bronchial epithelium [28–30]. After 16 weeks of NTCU treatment, there was a distinct change in the mitotic cell type from CCSP^+^ to K5^+^ cells (Fig 7 C and 7 D). The contribution of CCSP^+^ cells to the mitotic pool was less than 1% either for vehicle treated or any time after NTCU treatment (Fig 7D). In contrast, 11.5 ± 1.4% of newly expressed K5^+^ cells proliferated at 25 weeks and 18.6 ± 2.5% of these cells proliferated at 32 weeks (Fig 7D). It is important to note that the change in the mitotic cell type was limited to the larger airways where NTCU-induced dysplasia was readily observed. There was no significant change in the mitotic index of smaller airways where normal distribution of CCSP^+^ and ACT^+^ cells were maintained. These results demonstrate that the K5^+^ basal cells that populated mouse bronchial epithelium post NTCU are highly proliferative.

## Discussion

**Fig 8:**
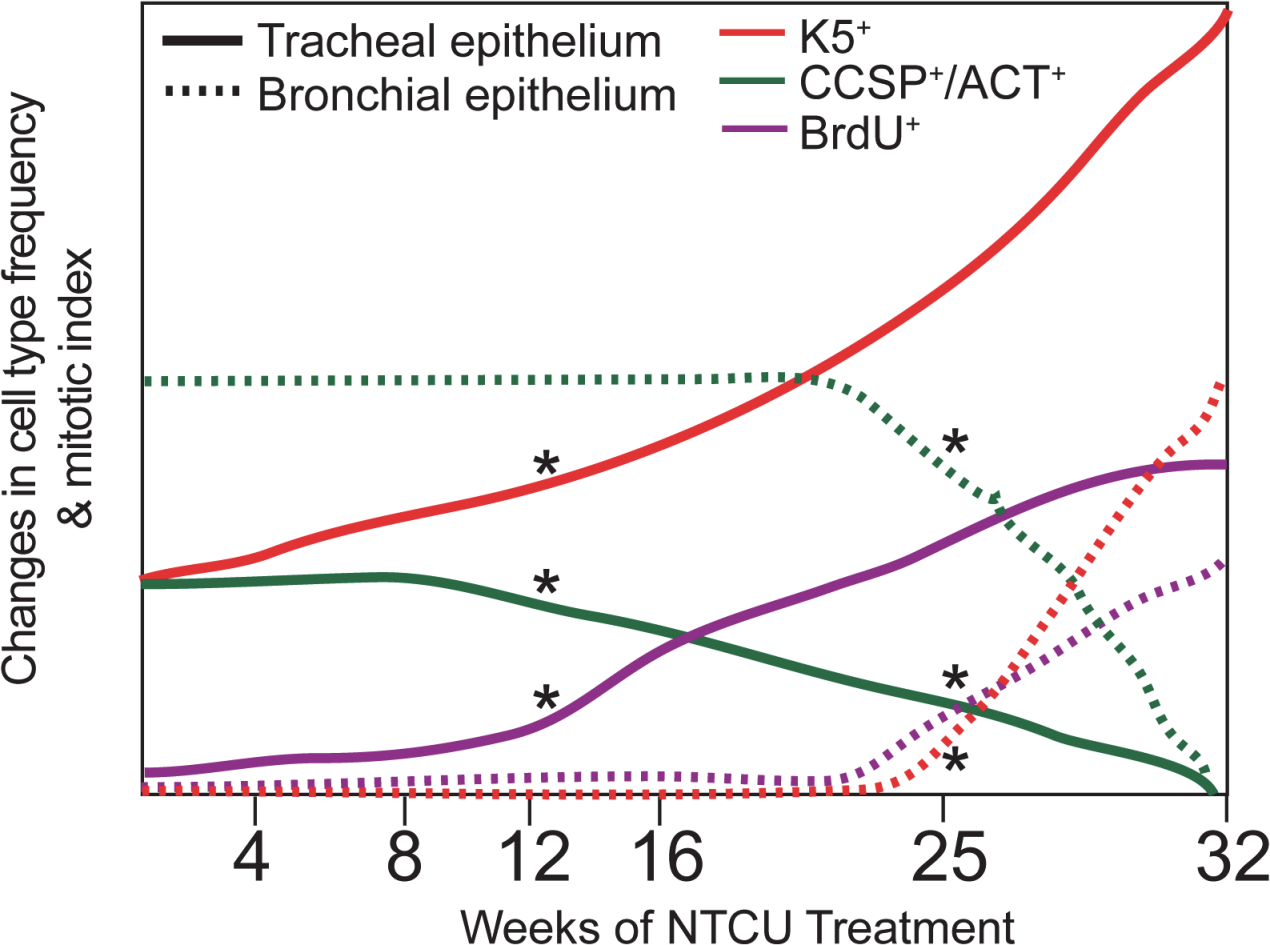
Summary of events post NTCU. A graphical representation of change in cell type frequencies in the trachea (solid line) and bronchial epithelium (dashed line) during NTCU time-course. Time points where changes are statistically significant are shown by * symbol.

In this study, we utilize the NTCU model to determine the cellular processes that precede endobronchial squamous dysplasia and SCC. This analysis led to the following results (summarized in Fig 8): 1) Dysplastic changes in the tracheal epithelium happened significantly earlier than in the bronchial epithelium; 2) Tracheal dysplasia involved increased frequencies of K5, K14 and, p63 expressing basal cells and loss of Club (CCSP^+^) and ciliated (ACT^+^) cells; 3) Chronic NTCU treatment converts pseudostratified tracheal epithelium to an involucrin expressing stratified epithelium; 4) NTCU-induced tracheal dysplasia caused increased proliferation of tracheal basal cells; 5) Dysplasia of the bronchial epithelium was first observed at 25 weeks, and involved appearance of K5^+^/K14^+^ cells and the loss of resident CCSP^+^ and ACT^+^ cells; and, 6) NTCU exposure altered bronchial mitotic pool from CCSP^+^ to K5^+^ cells.

The basal cell containing regions of the normal mouse airways are restricted to the trachea and proximal bronchi [13]. However, injury mediated depletion of bronchial CCSP^+^ cells recruits tracheal basal cells to participate in repair processes [28]. Also, H1N1 influenza virus mediated lung injury induces the appearance of K5^+^ cells in the distal lung epithelium [31]. These studies suggest that depending on the injury, basal cells can participate in bronchial and alveolar repair. In contrast to these acute injury studies, NTCU-mediated chronic injury showed a steady increase in the frequency of K5^+^ cells (initially in the trachea but later in the bronchial epithelium). Importantly, appearance of nascent K5^+^ cells in the bronchial epithelium was accompanied by the loss of resident CCSP^+^ and ACT^+^ cells. These results suggest unique cellular responses in acute and chronic injury, and may help model the development of human dysplasia that arises with chronic injury (for example, cigarette smoke exposure).

Repeated NTCU exposure also increased the numbers of K5^+^/K14^+^ cells, first in the trachea, and then in the bronchial epithelium. Previous studies from our group had shown that tracheal injury increased frequencies of these dual-positive cells, and this reverts to normal after epithelial repair [15]. Therefore, K5^+^/K14^+^ cells are termed as reparative basal cells (15). In human dysplasia, persistent increase in K14^+^ cells is associated with a poor prognosis of SCC [32]. Constitutive expression of human K14 gene in mouse lung induces premalignant lesions and squamous differentiation [33]. In stratified squamous epithelium, increased K14 expression promoted proliferation and knockdown of K14 significantly decreases tumorigenicity [34]. These results, in combination with our findings that increase in K5^+^/K14^+^ cells in the trachea occurred long before bronchial lesions developed; suggest that these cells might initiate the NTCU-induced injury process.

In contrast to the K14 upregulation in all K5^+^ cells; NTCU treatment increased expression of p63 only in a subset of K5^+^ cells. In the bronchial epithelium of 25 weeks NTCU treated mice, 5.1 ± 1.3% of K5^+^ cells co-expressed p63, and this increased to 25.2 ± 3.6% at 32 weeks. Several studies have described K5^+^/p63^+^ cells as the regenerative stem cells for both tracheal and distal lung epithelium [18, 31, 35]. Increase in this regenerative cell population after NTCU treatment suggests that aberrant epithelial repair could play a role in development and progression of bronchial dysplasia.

Interestingly, K5^+^/K14^+^ basal cells exhibited a uniform distribution pattern throughout the dysplastic epithelium whereas both p63^+^ and NGFR^+^ basal cells were limited to the epithelial layer adjacent to the basement membrane. Our previous studies of mouse and human airway epithelium showed phenotypic and functional heterogeneity within the basal cell population [36, 37]. Analyses of human airway dysplasia revealed lower numbers of Ki67^+^ cells in the epithelial layer that are adjacent to the basement membrane compared to non-adjacent layers [38]. We anticipate that functional analyses of basal cell subsets from dysplastic epithelium would help to identify the cell type involved in disease progression.

Two-progenitor populations, K5^+^ basal and CCSP^+^ secretory cells are required to maintain steady state tracheal epithelium. Injury-mediated depletion of CCSP^+^ progenitors led K5^+^ progenitors to reconstitute the epithelium by generating all the cell types of the tracheal epithelium [22, 39]. Subsequently, we showed that a subset of K5^+^ cells functions as the stem cells, these cells remain quiescent at steady state but proliferate after tracheal injury and participate in repair [40]. However, in response to repetitive NTCU injury, tracheal stem cells failed to re-establish a native epithelium. Functional analyses of tracheal stem cells at different time points of NTCU treatment would provide critical information about the role of K5^+^ basal stem cells during the development and progression of dysplasia.

In contrast to the trachea, CCSP^+^ cells are the major mitotic cell type for bronchial epithelium of normal mice [29, 30]. In agreement with these results we also showed that BrdU^+^ mitotic cells in the bronchial epithelium from vehicle-treated mice co-express CCSP. However, in NTCU-induced dysplasia the mitotic cells were K5^+^ basal cells. These results suggest that the basal cells play an essential role in the development of dysplasia and its progression to SCC.

Collectively, our results showed an early response of tracheal basal cells in NTCU-induced dysplasia and SCC. Using a time-course analysis of NTCU treatment we discovered a novel series of histologic and phenotypic changes in the tracheal epithelium that have not been previously reported. An improved understanding of these cellular mechanisms, particularly the contribution of tracheal basal cells in the development of distal airway dysplasia, could provide new targets for future chemopreventive efforts.

## Supporting Information

S1 Fig Basal cell containing region of the mouse respiratory tree. The mouse respiratory epithelium is divided into the tracheal, bronchial, bronchiolar and alveolar segments. Basal cells (yellow) populate the tracheal epithelium and proximal bronchi. This is also the cartilaginous area of the mouse respiratory tree (blue rings). Green arrow shows the main axial pathway, where NTCU-induced squamous dysplasias were observed.
(TIF)

S2 Fig 20 mM NTCU caused squamous dysplasia of the mouse bronchial epithelium and lung SCC. **(A)** The graph of mean body weight of mice treated with vehicle or with NTCU over time. **(B)** Percent survival for vehicle and NTCU treated mice. **(C)** Invasive SCC expressed basal cell markers K5 (green), **(D)** K14 (red), **(E)** transcription factor p63 (nuclear staining, red arrow) and **(F)** did not express thyroid transcription factor 1 (TTF1). SSC was developed from high-grade dysplasia shown by white arrows in C & D. **(G)** NTCU-induced dysplasia occurs in the bronchial epithelium along the main axial pathway (blue arrow). **(H)** Amplified image of the boxed area showing high-grade dysplasia. Original magnifications are mentioned in each panel. Representative images from n = 10 each of vehicle and NTCU treated mice.
(TIF)

S3 Fig NTCU-induced dysplasia is associated with the loss of CCSP-ACT and appearance of K5-expressing cells. Adjacent serial sections of lung tissues from 32-weeks NTCU treated mice, **(A)** Tissue stained with CCSP (green) and ACT (red), and **(B)** with K5 (green). White broken-lines outlined the small airway that maintained normal CCSP/ACT staining, whereas the dysplastic epithelium is populated only with K5^+^ cells. Arrows show the distinction between the normal and dysplastic region. Representative pictures from 10 sections/mouse from a total of 10 NTCU treated mice. DAPI staining (blue) in all images indicates nuclei and scale bar is indicated in each panel.
(TIF)

S4 Fig Expression of K5^+^ cells in the mouse bronchial epithelium. **(A)** Presence of K5 (green) expressing basal cells in the bronchial epithelium of 32 weeks NTCU treated mice. **(B)** Amplified image of the boxed area in **A** shows the presence of flat atypia and dysplasia next to each other. (*) under the epithelium shows infiltrating immune cells. DAPI staining (blue) in all images indicates nuclei and scale bar is indicated in each panel. Representative images from n = 10 mice.
(TIF)

S5 Fig Time-dependent changes in tracheal histology. H & E stained tracheal sections from **(A)** 4 weeks, **(B)** 8 weeks, **(C)** 16 weeks, and **(D)** 25 weeks NTCU-treated mice. Representative images from 5–7 mice per time point are shown here.
(TIF)

S6 Fig Morphometric analyses of p63, NGFR and DAPI. Quantification of tracheal **(A)** p63, **(B)** NGFR and **(C)** DAPI during the time-course of NTCU exposure. n = 3–4 tracheas were used per time-point.
(TIF)

S7 Fig TUNEL staining of tracheal epithelium. **(A)** Positive control showing TUNEL positive cells (green nuclei), (**B**) 16 weeks, (**C**) 25 weeks and (**D**) 32 weeks NTCU treated tracheas. TUNEL^+^ cells (green arrows) were detected under the epithelium but not on the epithelium. Representative images from n = 3 tracheas from each time.
(TIF)
