# Supplementary figures and images for: Tracheal Dysplasia Precedes Bronchial Dysplasia in Mouse Model of N-Nitroso Trischloroethylurea Induced Squamous Cell Lung Cancer
Source: PLoS One. 2015 Apr 10;10(4):e0122823. doi: 10.1371/journal.pone.0122823 (PMC4393296; doi:10.1371/journal.pone.0122823)

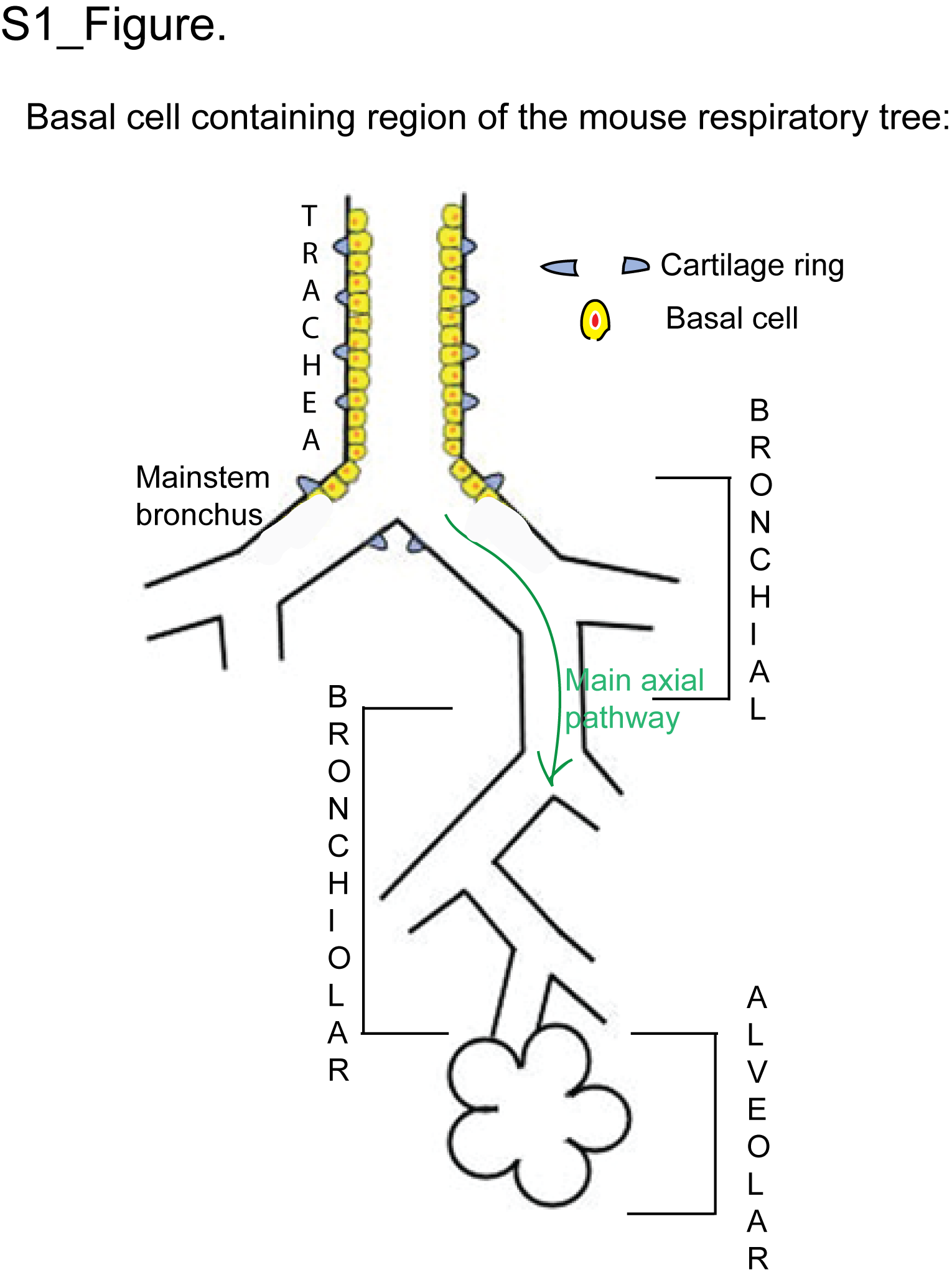

Supplement: S1 Fig — The mouse respiratory epithelium is divided into the tracheal, bronchial, bronchiolar and alveolar segments. Basal cells (yellow) populate the tracheal epithelium and proximal bronchi. This is also the cartilaginous area of the mouse respiratory tree (blue rings). Green arrow shows the main axial pathway, where NTCU-induced squamous dysplasias were observed. (TIF) [file pone.0122823.s001.tif]

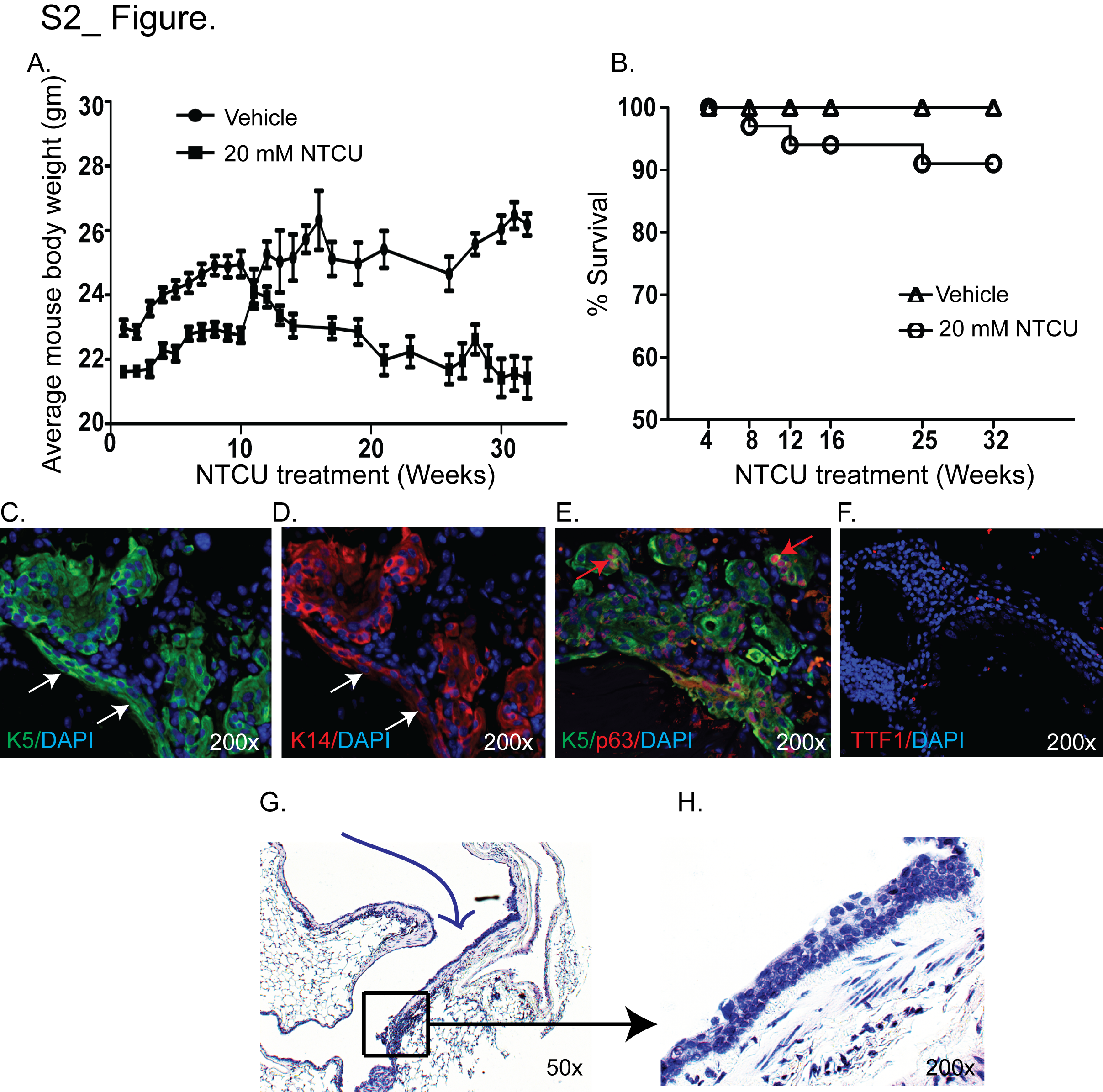

Supplement: S2 Fig — (A) The graph of mean body weight of mice treated with vehicle or with NTCU over time. (B) Percent survival for vehicle and NTCU treated mice. (C) Invasive SCC expressed basal cell markers K5 (green), (D) K14 (red), (E) transcription factor p63 (nuclear staining, red arrow) and (F) did not express thyroid transcription factor 1 (TTF1). SSC was developed from high-grade dysplasia shown by white arrows in C & D. (G) NTCU-induced dysplasia occurs in the bronchial epithelium along the main axial pathway (blue arrow). (H) Amplified image of the boxed area showing high-grade dysplasia. Original magnifications are mentioned in each panel. Representative images from n = 10 each of vehicle and NTCU treated mice. (TIF) [file pone.0122823.s002.tif]

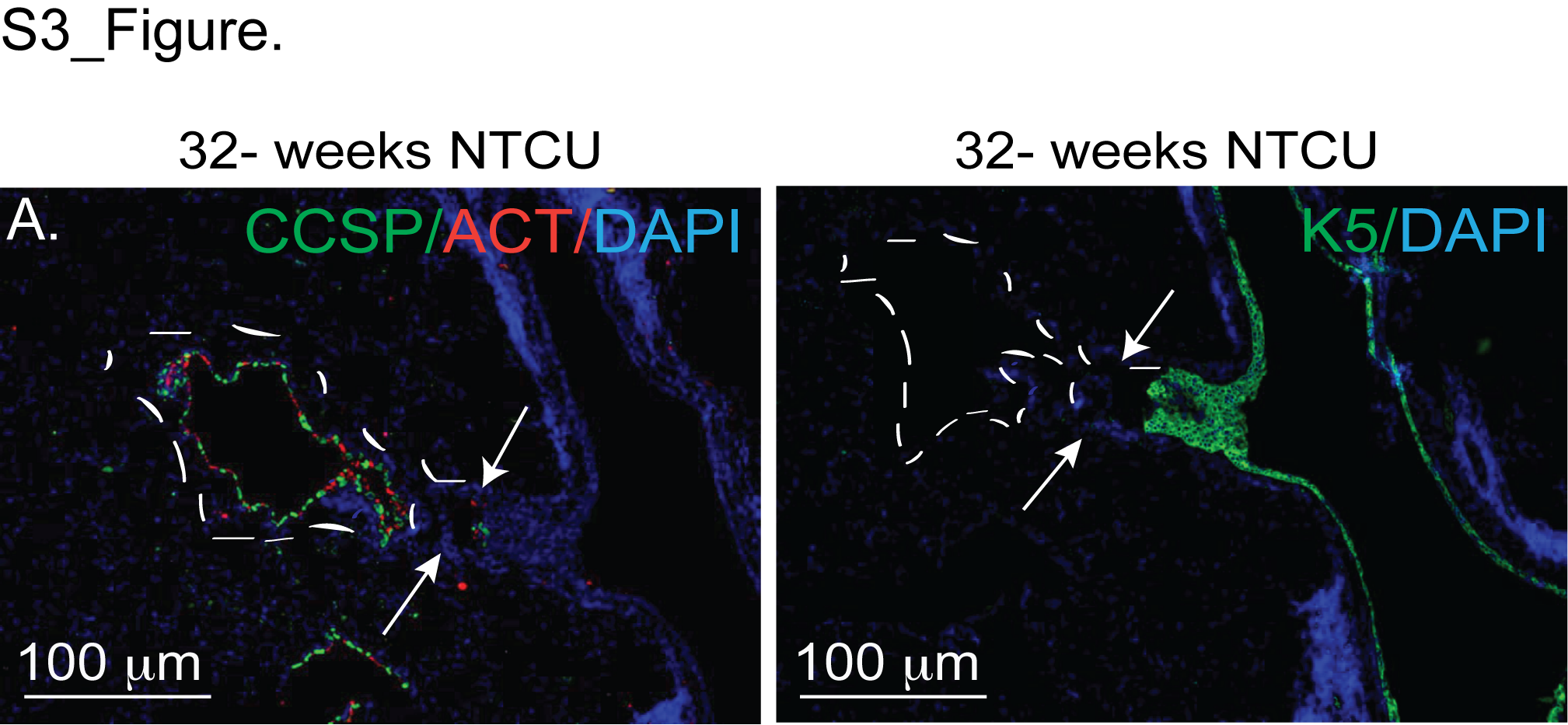

Supplement: S3 Fig — Adjacent serial sections of lung tissues from 32-weeks NTCU treated mice, (A) Tissue stained with CCSP (green) and ACT (red), and (B) with K5 (green). White broken-lines outlined the small airway that maintained normal CCSP/ACT staining, whereas the dysplastic epithelium is populated only with K5+ cells. Arrows show the distinction between the normal and dysplastic region. Representative pictures from 10 sections/mouse from a total of 10 NTCU treated mice. DAPI staining (blue) in all images indicates nuclei and scale bar is indicated in each panel. (TIF) [file pone.0122823.s003.tif]

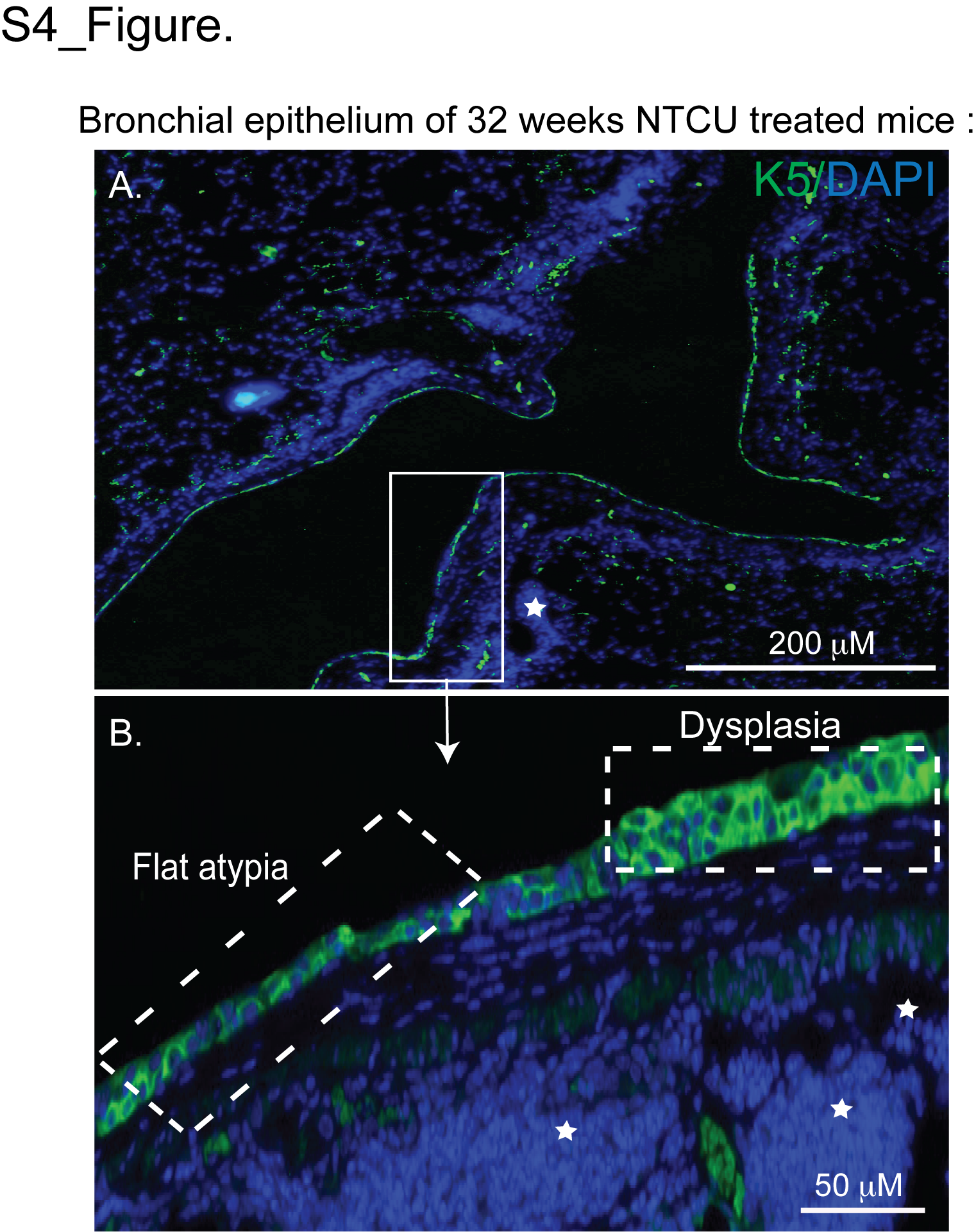

Supplement: S4 Fig — (A) Presence of K5 (green) expressing basal cells in the bronchial epithelium of 32 weeks NTCU treated mice. (B) Amplified image of the boxed area in A shows the presence of flat atypia and dysplasia next to each other. (*) under the epithelium shows infiltrating immune cells. DAPI staining (blue) in all images indicates nuclei and scale bar is indicated in each panel. Representative images from n = 10 mice. (TIF) [file pone.0122823.s004.tif]

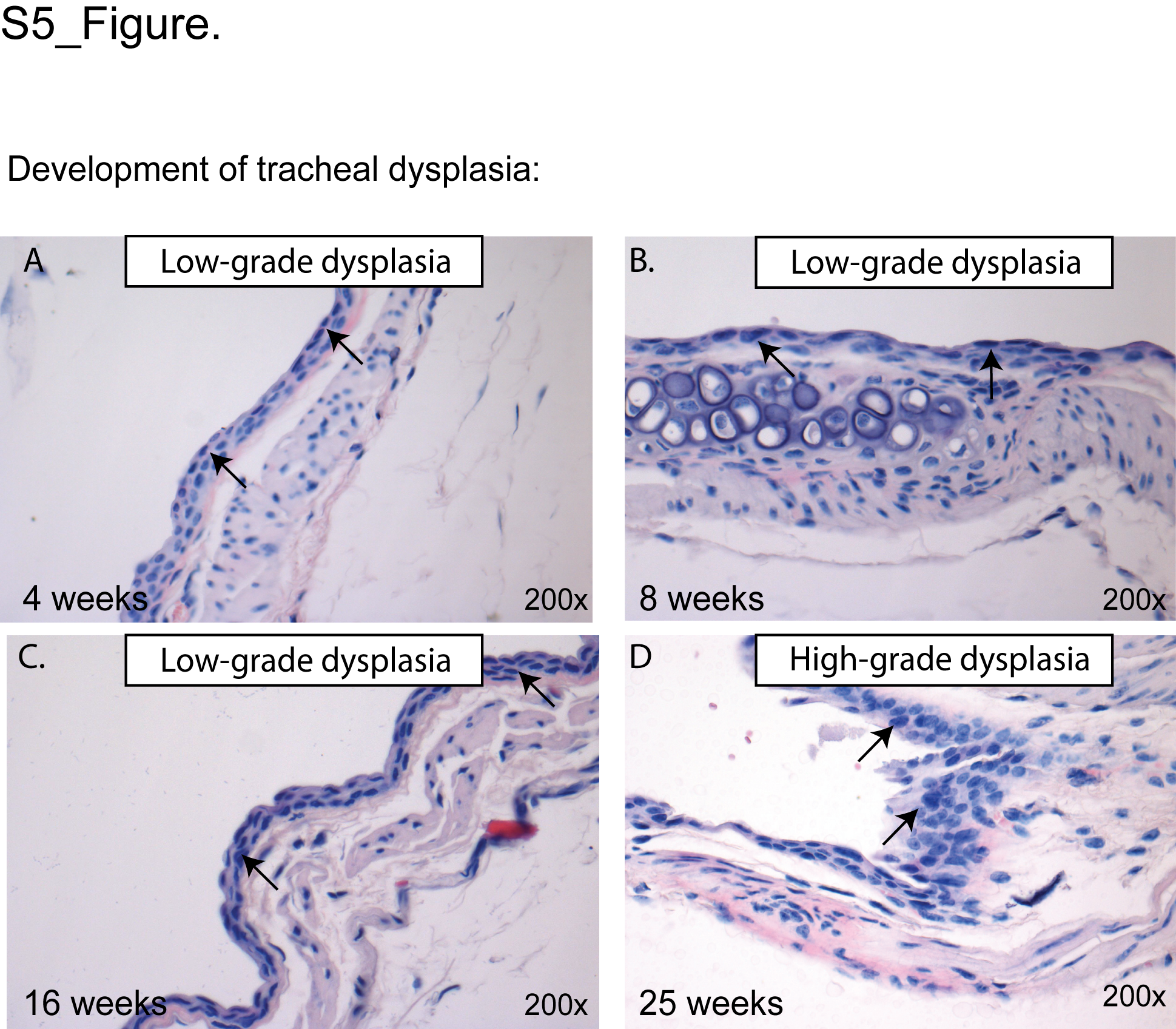

Supplement: S5 Fig — H & E stained tracheal sections from (A) 4 weeks, (B) 8 weeks, (C) 16 weeks, and (D) 25 weeks NTCU-treated mice. Representative images from 5–7 mice per time point are shown here. (TIF) [file pone.0122823.s005.tif]

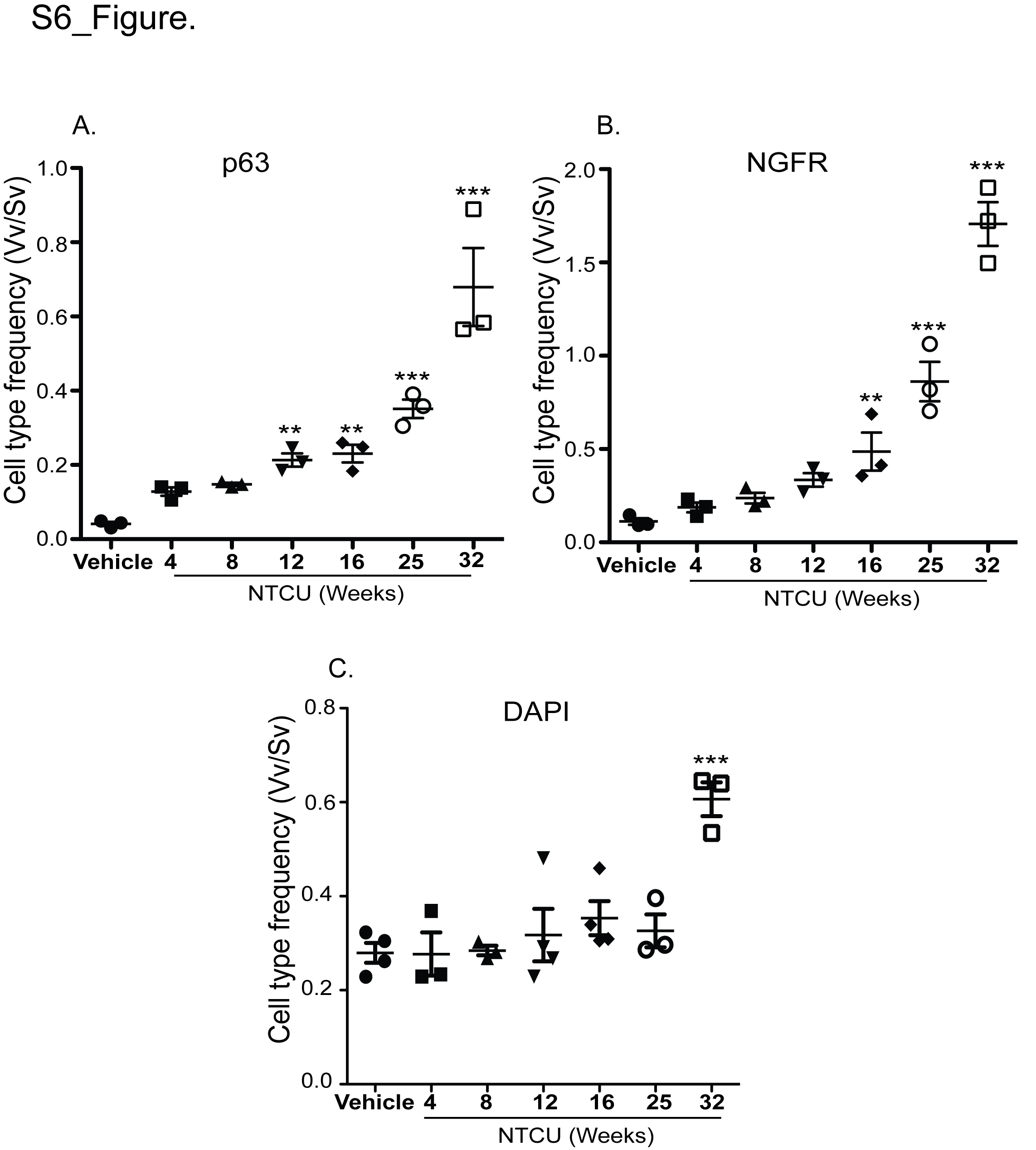

Supplement: S6 Fig — Quantification of tracheal (A) p63, (B) NGFR and (C) DAPI during the time-course of NTCU exposure. n = 3–4 tracheas were used per time-point. (TIF) [file pone.0122823.s006.tif]

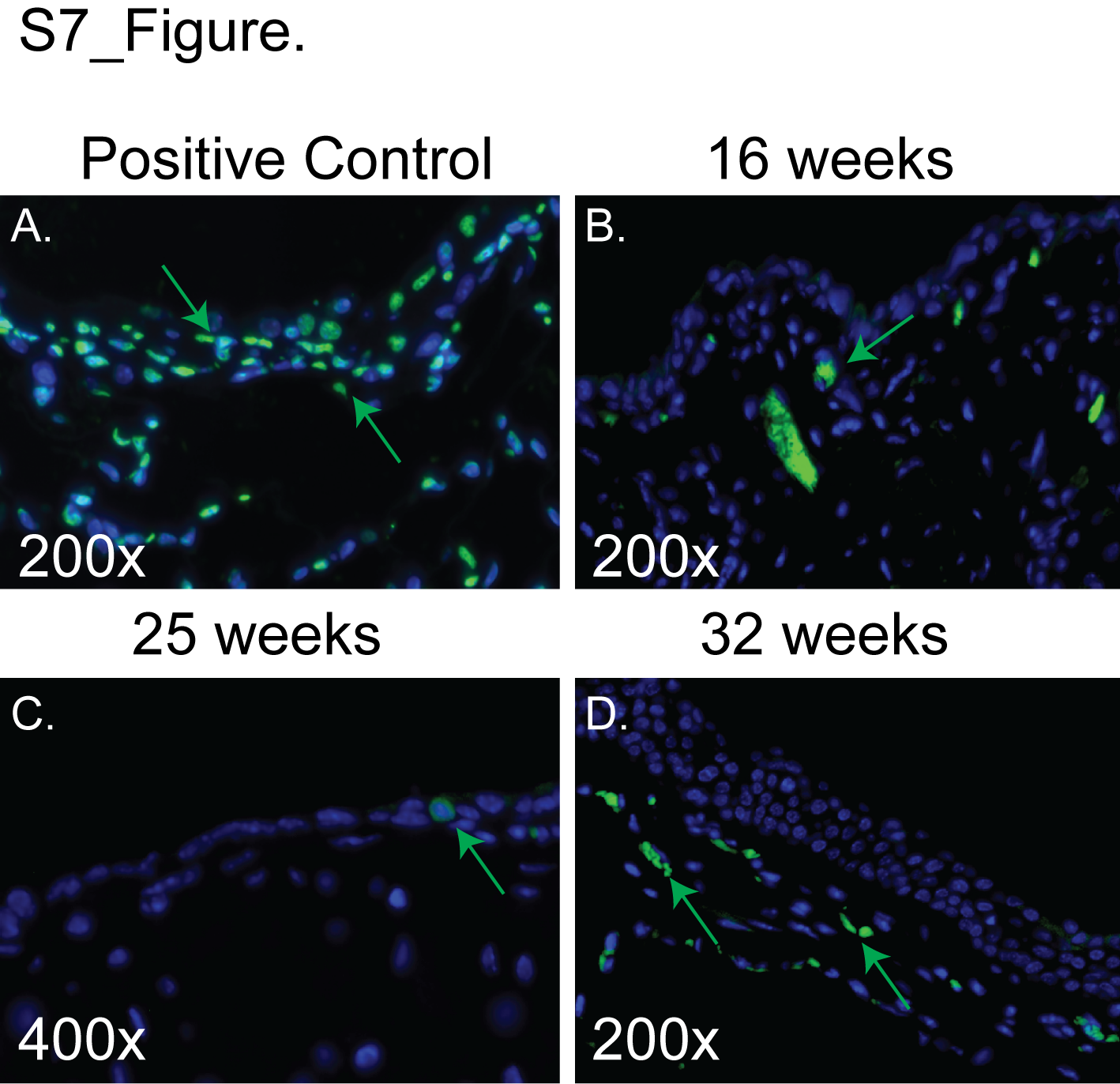

Supplement: S7 Fig — (A) Positive control showing TUNEL positive cells (green nuclei), (B) 16 weeks, (C) 25 weeks and (D) 32 weeks NTCU treated tracheas. TUNEL+ cells (green arrows) were detected under the epithelium but not on the epithelium. Representative images from n = 3 tracheas from each time. (TIF) [file pone.0122823.s007.tif]
